# Identification of *O*-glycosylated Proteins That Are Aberrantly Excreted in the Urine of Patients with Early Stage Ovarian Cancer

**DOI:** 10.3390/ijms14047923

**Published:** 2013-04-11

**Authors:** Alan Kang-Wai Mu, Boon-Kiong Lim, Onn Haji Hashim, Adawiyah Suriza Shuib

**Affiliations:** 1 Institute of Biological Sciences, Faculty of Science, University of Malaya, Kuala Lumpur 50603, Malaysia; E-Mail: alanmukangwai@yahoo.com; 2 Department of Obstetrics and Gynaecology, Faculty of Medicine, University of Malaya, Kuala Lumpur 50603, Malaysia; E-Mail: limbk@ummc.edu.my; 3 Department of Molecular Medicine, Faculty of Medicine, University of Malaya, Kuala Lumpur 50603, Malaysia; E-Mail: onnhashim@um.edu.my; 4 University of Malaya Centre for Proteomics Research, Faculty of Medicine, University of Malaya, Kuala Lumpur 50603, Malaysia

**Keywords:** ovarian cancer, urine, biomarker, *O*-glycan, champedak galactose binding lectin, two-dimensional gel electrophoresis, proteomics

## Abstract

Cancer is known to induce or alter the *O*-glycosylation of selective proteins that may eventually be excreted in the patients’ urine. The present study was performed to identify *O*-glycosylated proteins that are aberrantly excreted in the urine of patients with early stage ovarian cancer (OCa). These urinary glycoproteins are potential biomarkers for early detection of OCa. In this study, urinary proteins of patients with early stage OCa and age-matched OCa negative women were subjected to two-dimensional gel electrophoresis and detection using a lectin that binds to the *O*-glycosylated proteins. Our analysis demonstrated significant enhanced expression of clusterin and leucine-rich alpha-2-glycoprotein, but lower levels of kininogen in the urine of the OCa patients compared to the controls. The different altered levels of these urinary glycoproteins were further confirmed using competitive ELISA. Our data are suggestive of the potential use of the aberrantly excreted urinary *O*-glycosylated proteins as biomarkers for the early detection of OCa, although this requires further validation in a large clinically representative population.

## 1. Introduction

Ovarian cancer (OCa) is the fifth most prevalent cause of cancer death in women [1]. The five-year survival rate of patients diagnosed at an advanced stage is approximately 30%, and this increases to more than 90% if the patients were diagnosed at stage I [2]. Currently, diagnosis of OCa is mainly reliant on imaging methods and assessment of tumor markers. However, CA125, the main tumor marker used in the diagnosis of OCa, is also elevated in numerous other cancers and benign conditions [3]. In addition, the tumor marker is not adequately sensitive for early detection, as many women with early stage OCa have normal CA125 levels [4]. In view of these limitations, there is a need to identify new biomarkers that are more specific and sensitive, which can improve diagnosis of early stage OCa and, hence, the survival rates of the patients.

The collection of human urine is more convenient and non-invasive compared to serum, plasma or tissue specimens. This makes it one of the preferred sample choices for diagnostic purposes. Attempts to identify protein biomarkers in the urine are gaining popularity, particularly with the advancement of proteomics technology. This includes profiling of the urine protein samples by two-dimensional gel electrophoresis (2-DE) and identification of the proteins that are aberrantly expressed using MALDI-ToF or other mass spectrometry techniques and searches of databases. Research using these approaches is not restrictive to the screening for biomarkers, but also improves understanding of the disease and, perhaps, the management and treatment of cancer.

*O*-glycosylation is an enzymatic reaction in which a glycan moiety is attached to the serine or threonine residues of a protein. The attached *O*-glycans greatly influence the stability and tertiary structures of the glycoproteins and, hence, their functions in the biological system. Many serum *O*-glycosylated proteins have been previously implicated in cancer [5–7], either in the form of their altered *O*-glycan structures or their levels. These findings not only provide meaningful information about cancer development, but the glycoproteins that are modified may also be exploited for their potential use as cancer biomarkers.

The aim of the present study was to screen for potential *O*-glycosylated protein biomarkers in the urine of patients with OCa using the lectin-based proteomics approach. Urine samples of untreated and newly diagnosed patients with early stage OCa, as well as OCa negative women subjects, were initially subjected to 2-DE separation. This was followed by Western blotting and detection using an enzyme-conjugated champedak galactose binding (CGB) lectin. The CGB lectin, which binds specifically to the *O*-glycan structures [8,9], was used to probe for the *O*-glycans of glycoproteins. The *O*-glycosylated proteins that were found to be aberrantly excreted in the urine of the OCa patients were then identified by mass spectrometry and database search.

## 2. Results

### 2.1. Image Analysis of *O*-glycosylated Protein Profiles

**Figure 1:**
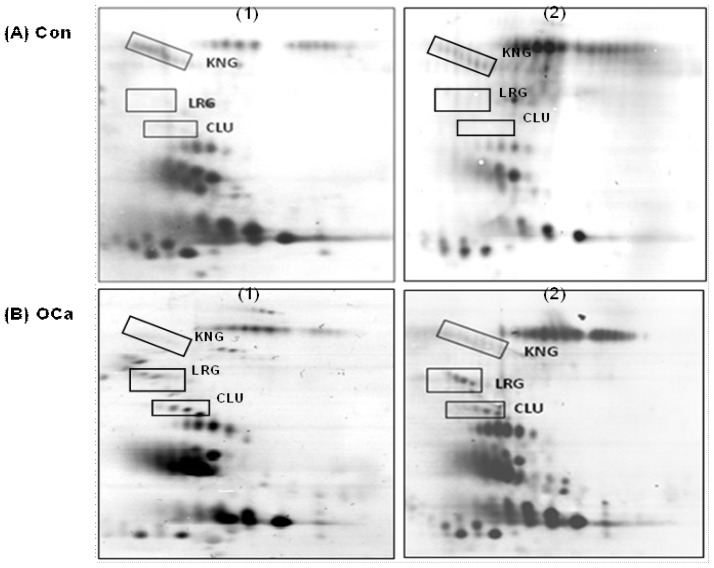
Two-dimensional gel electrophoresis (2-DE) *O*-glycosylated protein profiles of controls and ovarian cancer (OCa) patients. Panels **A** and **B** demonstrate representative 2-DE urinary *O*-glycosylated protein profiles of controls and OCa patients, respectively. Protein clusters that were labeled (clusterin (CLU), leucine-rich alpha-2-glycoprotein (LRG) and kininogen (KNG)) are those found to be aberrantly excreted in the urine of the patients. The acidic side of the gel is to the left, and relative molecular mass is from the top.

Profiles of *O*-glycosylated proteins of the OCa patients and controls were generated using enzyme-conjugated CGB lectin to bind to the urinary proteins that had been separated by 2-DE and transferred onto nitrocellulose membrane. Figure 1 demonstrates representatives of the *O*-glycosylated protein profiles of control subjects (Panel A) and OCa patients (Panel B). The 2-DE profiles of the patients and controls appeared comparable, except for three clusters of urinary *O*-glycosylated proteins. Two of the glycoprotein clusters appeared to be exclusively present in the profiles of the OCa patients, while the intensity of one protein cluster was consistently reduced compared to that of the control subjects.

Subjecting the two upregulated *O*-glycosylated proteins to mass spectrometric (MS) analysis and database search identified the glycoproteins as those of clusterin (CLU) and leucine-rich alpha-2-glycoprotein (LRG) (Table 1). Data for the identification of the kininogen (KNG) spot cluster had been previously reported [7]. Analysis of the volumes of the proteins showed that the levels of LRG and CLU in the OCa patients were both 17-fold higher than the controls, while the level of the patients’ KNG was six-fold lower than the controls (Table 2).

### 2.2. Competitive ELISA

**Figure 2:**
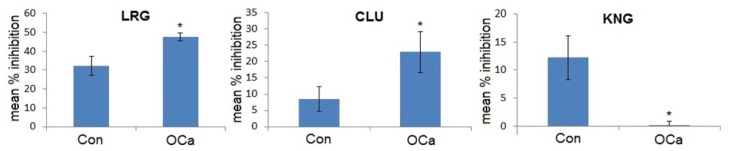
Mean percentage of inhibition of urinary LRG, CLU and KNG in controls (Con) and patients with OCa. Analysis was performed by competitive ELISA. The concentrations of LRG and CLU in the urine samples of OCa patients were significantly higher than those of the controls, while KNG was significantly lower in the OCa patients. The concentrations of proteins present in the samples were proportional to the percentage inhibition of substrate hydrolysis. Asterisk denotes the statistical significance with *p <* 0.05.

Competitive ELISA was performed using monoclonal antisera against LRG, CLU and KNG to validate the altered levels of the urinary proteins that were detected in the patients with OCa (*n =* 9) compared to the control subjects (*n =* 11). Figure 2 demonstrates the mean percentage of inhibition of LRG, CLU and KNG that were tested in respective groups of samples. The expression of LRG and CLU was significantly enhanced, whilst KNG was found to be decreased in almost all of the urine samples of the OCa patients compared to the controls.

## 3. Discussion

The finding of altered levels of glycoproteins, which are potential biomarkers, may not only improve diagnosis and the survival rates of patients suffering from OCa, but also provide more insights into the molecular mechanisms of the disease. In the present study, the significant increased levels of LRG and CLU and decreased levels of KNG were detected in the urine samples of OCa patients compared to those of the controls when image analysis was performed on the CGB lectin-detected *O*-glycosylated proteins that were separated by 2-DE and transferred onto nitrocellulose membranes. The significant altered expression of the three urinary *O*-glycosylated proteins in the OCa patients was further confirmed using competitive ELISA.

The altered levels of urinary LRG and CLU that were detected in the present study have been previously observed in the sera of patients with OCa [10]. Hence, it is reasonable to postulate that the increase of both the glycoproteins in the urine of the OCa patients’ may be reflective of changes that had occurred in their serum.

LRG is a secreted protein with regions containing leucine-rich repeats. It is mainly produced by the liver cells in response to cancer, but its synthesis by OCa cells has also been documented [11]. The actual functions of LRG are still unknown, although the glycoprotein has been associated with cell adhesion, granulocytic differentiation, cell migration, cell survival, protein interaction and signal transduction [12]. The *O*-glycosylated protein was also proposed to be involved in the acute-phase response, since its level was reported to be elevated in patients with microbial infection, as well as those afflicted with several different cancers, including OCa [11].

Increased levels of CLU have been previously observed in the sera of patients with OCa, as well as those with other types of cancers [10,13,14]. CLU, also known as apolipoprotein J, has been implicated in diverse physiological functions. Its expression was previously reported to be associated with regulation of the cell cycle, cell damage recovery, cell survival, senescence, tumorigenesis and apoptosis [15]. Our present data demonstrated increased levels of CLU in the urine of the OCa patients.

KNG is a protein containing two isoforms produced by alternative mRNA splicing [16]. Based on the molecular weight of the 2-DE separated glycoprotein that was observed on the membrane, the KNG that was detected in the present study was most likely its light chain component that is also known to be *O*-glycosylated. The decreased in the levels of KNG in the urine of OCa patients has been previously reported, and this was suggested to act as compensation to the mechanisms of angiogenesis and cell proliferation, because KNG was shown to inhibit angiogenesis, as well as the proliferation of endothelial cells [17]. In addition to the data of these studies, there have also been a number of similar reports on the decreased levels of KNG in patients with cervical cancer [5], breast cancer [14] and gastrointestinal cancer [18].

## 4. Experimental Section

### 4.1. Urine Samples and Processing

Urine samples from newly diagnosed patients with stages I and II OCa (*n =* 9) were collected at the Gynecological Clinic, University of Malaya Medical Centre, Kuala Lumpur, prior to any treatment or surgery. Table 3 demonstrates the clinical data of the OCa patients involved in the study. The processing of the samples was performed as previously described [9]. Patients with any other diseases aside from OCa were excluded from the study.

Control urine samples (*n* = 11, ages of 30–75) were obtained randomly from OCa negative women. All samples were obtained with the subjects’ consent and approval granted by the Ethical Committee of UMMC in accordance to the ICH GCP guideline and the declaration of Helsinki.

### 4.2. Isolation of CGB Lectin

CGB lectin was purified as previously described [7,8]. Briefly, crude extracts of the seeds of champedak were prepared by stirring of homogenized seeds in PBS, pH 7.4, overnight, and subjecting the supernatant to 60% ammonium sulfate precipitation. The crude extract was then subjected to immobilized galactose affinity chromatography, and elution of the CGB lectin was performed using 0.8 M galactose. Purity of the eluted lectin was confirmed by SDS-PAGE. The purified lectin was then conjugated to horse radish peroxidase before it was used in Western blot analysis.

### 4.3. Two-Dimensional Electrophoresis

Urinary proteins of patients and controls were resolved using 2-DE, which was performed as previously described [19]. Briefly, 300 μg of freeze dried urine samples were subjected to isoelectric focusing in 11 cm rehydrated precast Immobiline Drystrips pH 3–10 using the IPGphor 3 equipment (GE Healthcare Biosciences, Uppsala, Sweden). This was followed by electrophoretic separation of the focused protein samples in the second dimension in 12.5% polyacrylamide gels in the presence of SDS.

### 4.4. Western Blotting and Lectin Detection

Glycoproteins were transferred from 2-DE gels onto nitrocellulose membranes (0.45 μm) using the NovaBlot Kit of Multiphor II Electrophoresis system (GE Healthcare Bioscience, Uppsala, Sweden) at 0.8 mA/cm^2^ for 1 h. The membrane was blocked with 3% w/v gelatin in Tris-buffered saline Tween-20 (TBST), pH 7.5, for 1 h at room temperature and washed three times with the same buffer. Detection of transferred *O*-glycosylated urinary proteins was performed using horseradish peroxidase-conjugated CGB lectin. Blots were developed using a metal enhanced diaminobenzidine (DAB) substrate kit for horseradish peroxidase (Pierce, Rockford, IC, USA).

### 4.5. Image Analysis

The lectin blots were scanned using the ImageScanner III (GE Healthcare Bioscience, Uppsala, Sweden). Image analysis was restricted to protein spot clusters that appeared consistently within each group of urine samples. The levels of proteins in each sample were calculated as a percentage of volume contribution (%vol). This refers to the volume percentage of a protein taken against the total spot volume of all proteins. The %vol was used in order to eliminate possible variations in the total amounts of proteins analyzed. The Student’s *t*-test was used to analyze the significance difference of the %vol of a spot/protein cluster between the controls and OCa patients. A *p*-value of less than 0.05 was initially considered significant. Correction of false positive discoveries was then carried out based on the method of Benjamini and Hochberg [20]. Consequently, a *p*-value of less than 0.022 was subsequently considered to be significant.

### 4.6. MALDI-ToF Mass Spectrometry

Identification of proteins was performed by mass spectrometry and database search. The 2-DE gels were silver stained according to the method of Shevchenko *et al.*[21]. In-gel digestion of protein plugs from 2-DE gels was carried out according to a previously described method [22]. On the other hand, on-membrane digestion was performed according to the method described by Mu *et al.*[7]. The MALDI mass spectrometry was performed using the Applied Biosystem 4800 Proteomics Analyzer. The MS/MS data were then submitted to the MASCOT search engine for protein identification. Proteins were considered positively identified, as described by Delahunty and Yates [23].

### 4.7. Competitive ELISA

The altered levels of LRG, CLU and KNG were validated using competitive ELISA, as described by Doustjalali *et al.*[13], with slight modifications. Wells of microtiter plates (Nunc, Denmark) were coated with 10 μg urinary protein in 0.05 M sodium bicarbonate buffer pH 9.6 overnight at 4 °C. The wells were rinsed extensively with 100 μL of PBST three times. This was followed by blocking with 100 μL of 0.5% gelatin in the same buffer for 30 min at room temperature. After blocking, the plates were washed, and the wells were filled with mouse anti-human LRG, CLU and KNG in the presence of controls’ (*n =* 11) and patients’ (*n =* 9) urine samples at a dilution of 1:1000 for 1 h at room temperature. Positive controls and blanks were prepared in the absence of urine samples and by addition of washing buffer, respectively. After incubation, each well was washed extensively with washing buffer. This was followed by the addition of 200 μL of horseradish peroxidase-conjugated secondary anti-mouse IgG at 1:5000 dilution. The plates were incubated for another 1 h at room temperature before they were washed extensively with washing buffer. One hundred μL of 3,3′,5,5′-tetramethylbenzidine substrate (Sigma Aldrich, St. Louis, MO, USA) was added to each well, and the plates were incubated for 15 min at room temperature in the dark. The reaction was terminated with the addition of 100 μL 2 M sulfuric acid. Absorbance values were read at 450 nm using an ELISA plate reader. The amounts of the respective proteins in the urine samples were determined based on the percentage of inhibition of substrate hydrolysis [24].

## 5. Conclusions

The present study identified the different altered expression of LRG, CLU and KNG in the urine of a group of patients with early stage OCa, which may be used as complementary biomarkers for early detection of the cancer. Our findings also emphasize the need for further epidemiological validation studies to assess the sensitivity and specificity of the potential urinary biomarkers for the early stage diagnosis of OCa.

## Figures and Tables

**Table 1:** Identification of aberrantly excreted urinary *O*-glycosylated proteins by mass spectrometry (MS).

Protein entry name	Protein name	Accession number #	Nominal mass (kDa)/p*I*	MOWSE protein score	Sequence coverage (%)
LRG	Leucine-rich alpha-2-glycoprotein	P02750	38/6.45	149	10
§ CLU	Clusterin	P10909	52/5.89	25	11

Protein entry names are from the UniProtKB database;

# Accession numbers are from the Mascot search engine;

§ On-membrane digestion.

**Table 2:** Relative expression of aberrantly excreted urinary *O*-glycosylated proteins.

*O*-Glycosylated protein	Mean %vol ± SEM		*p*	Fold changes

	Control	OCa		
LRG	0.120 ± 0.077	2.06 ± 0.298	0.000004	+17.17
CLU	0.105 ± 0.080	1.821 ± 0.356	0.0001	+17.34
KNG	10.135 ± 2.163	1.638 ± 0.505	0.004	−6.19

**Table 3:** Clinical data of OCa patients involved in the study.

OCa patient	Stage	Age	Late menopause	Previous pregnancy	Early puberty	Blood creatinine
1	I	48	No	Yes	No	Normal
2	I	56	No	Yes	No	Normal
3	I	62	No	Yes	No	Normal
4	I	70	No	Yes	No	Normal
5	I	81	No	Yes	No	Normal
6	II	46	No	Yes	No	Normal
7	II	54	No	Yes	No	Normal
8	II	68	No	Yes	No	Normal
9	II	47	No	Yes	No	Normal
